# New molecular evidence on the members of the genus *Ortholinea* (Cnidaria, Myxozoa) and the description of *Ortholinea hamsiensis* n. sp. infecting the urinary bladder of European anchovy *Engraulis engrasicolus* in the Black Sea

**DOI:** 10.1017/S0031182024000325

**Published:** 2024-04

**Authors:** Sevilay Okkay, Cem Tolga Gürkanlı, Yılmaz Çiftçi, Ahmet Özer

**Affiliations:** 1 Kocaeli University, Science and Arts Faculty, Department of Biology, 41001, Kocaeli, Türkiye; 2 Ordu University, Fatsa Faculty of Marine Sciences, Department of Fisheries Technology Engineering, 52400, Fatsa-Ordu, Türkiye; 3 Sinop University, Faculty of Fisheries and Aquatic Sciences, 57000, Sinop, Türkiye

**Keywords:** Black Sea, *Ortholinea*, Phylogeny, 18S rDNA

## Abstract

Members of the genus *Ortholinea* are among the worldwide distributed myxozoan parasites that mainly infect marine fish. In this study, a new myxosporean species, *Ortholinea hamsiensis* n. sp., was isolated from the urinary bladder of European anchovy *Engraulis engrasicolus* collected from the Sinop coasts of the Black Sea. The prevalence and density values of infection were 1.4% and 1–5 individuals in the field of view (1 + ), respectively. Mature myxospores are subspherical with slight tapering down to the less pronounced tip in the frontal view and subspherical in the sutural view. Myxospores measured 9.1 ± 0.25 (8.8–9.9) *μ*m in length, 9.2 ± 0.11 (8.9–9.4) *μ*m in thickness, and 8.4 ± 0.33 (8.2-9.1) *μ*m in width. Two polar capsules equal in size measured 3.1 ± 0.11 (3.0–3.3) *μ*m in length and 2.7 ± 0.11 (2.6–2.9) *μ*m in width. The polar tubule had 3–4 coils. Along with morphological peculiarities, the results of the 18S rDNA also revealed it to be a new species for science compared to the other species of the genus. In this study, another myxosporean species *O. gobiusi* was also detected in round goby *Neogobius melanostomus* with a prevalence of infection value of 4.8% and a density of 1–5 individuals in the field of view (1 + ). The present study also provided the first data of 18S rDNA of *O. gobiusi* from *N. melanostomus* and type species of the genus *O. divergens* from *Gobius niger* and the phylogenetic relationships of these species with other *Ortholinea* species have been revealed.

## Graphical abstract

**Figure:**
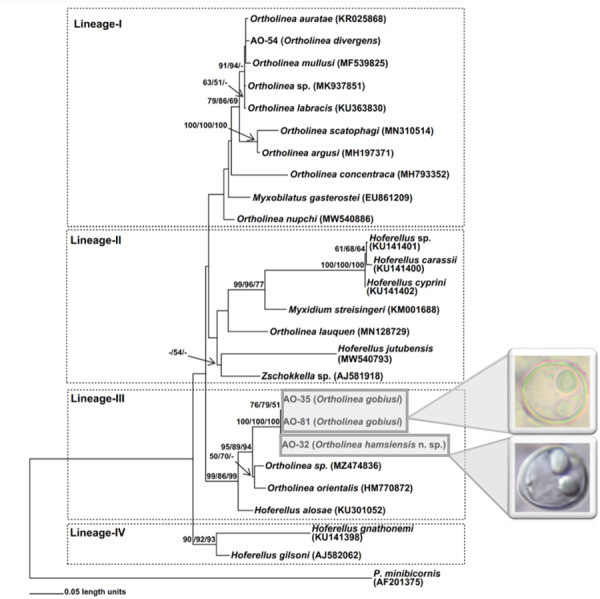

## Introduction

Myxozoans are the cnidarian parasites that have been reported from fish inhabiting freshwater, brackish water and marine environments (Lom and Dyková, 2006; Gürkanlı *et al*., 2018; Okkay and Özer, 2020). Description of myxozoan parasites is mainly based on myxospore morphology and according to this traditional criteria, species of the genus *Ortholinea* Shulman, 1962 have spherical or subspherical myxospores that are lightly flattened or tapered posterior parallel to the sutural plane, containing 2 polar capsules, subspherical or pyriform, and some species have surface stripes (Lom and Dyková, 2006). However, taxonomic placement based solely on morphological criteria has been proven to be artificial and molecular data of the SSU rRNA gene provided more accurate allocations in the taxonomy of myxozoan parasites (Rangel *et al*., 2017). *Ortholinea* is probably a genus known to have ancestors reviving marine habitats and it has recently been transferred from Ortholineidae to Myxobilatidae due to phylogenetic proximity (Karlsbakk *et al*., 2017). This genus is represented by 26 species worldwide including 4 species that have been reported from marine fishes in the Black Sea coasts of Türkiye (Lom and Dyková, 1992; Karlsbakk, 2001, Rangel *et al*., 2014, 2015, 2017; Özer *et al*., 2015*a*, 2015*b*; Gürkanlı *et al*., 2018; Shin *et al*., 2023). Most of the *Ortholinea* species have been generally reported in the urinary bladder, but rarely in the kidney, gallbladder, and gill tissues of their host fishes (Rangel *et al*., 2014, 2015, 2017; Gürkanlı *et al*., 2018). In a recent study, Okkay and Özer (2020), based on morphological criteria, reported *Ortholinea orientalis* from the urinary bladder of European anchovy, *Engraulis encrasicolus* (Linnaeus, 1758) and Pontic shad, *Alosa immaculata* Bennett, 1835, *Ortholinea divergens* from the kidney of grey wrasse, *Symphodus cinereus* (Bonnatterre, 1788) and *Ortholinea* sp. from the kidney of black goby, *Gobius niger* Linnaeus, 1758 collected from Sinop coasts of the Black Sea in Türkiye.

In the present study, we aimed to describe the phylogenetic peculiarities of above mentioned *Ortholinea* species and the description of possible new species among previously identified individuals based solely on myxospore morphology.

## Materials and methods

### Fish sampling and parasitological examination

In the present study, a total of 103 specimens of round goby *Neogobius melanostomus* (Pallas, 1814) were collected from a fisherman in the Sinop coast (42° 05′ 68″ N, 35° 10′ 55″ E) of the Black Sea, Türkiye, in the period September 2017–December 2019. Gills, fins, skin, urinary bladder, kidney, gall bladder, liver, intestine, smooth muscles and gonads of each fish species were investigated for the presence of *Ortholinea* parasites. Moreover, previously alcohol-preserved urinary bladder and kidney tissues of *Gobius niger*, *Symphodus cinereus* and *Engraulis encrasicolus* were re-investigated for *Ortholinea* myxospores by Okkay and Özer (2020). Myxospores of *Ortholinea* were examined and photographed with an Olympus microscope (BX53) equipped with a digital camera (DP50), at 400 ×  and 1000 ×  magnifications and Nikon (H550S) with DIC attachment at the Faculty of Fisheries and Aquatic Sciences in Sinop, Türkiye. Measurements were based on 20 fresh myxospores from *N. melanostomus* and 20 alcohol-preserved myxospores from *E. engrasicolus*, and morphological terminology and definitions are explained by Lom and Dyková (1992). All measurements are given with mean values ± standard deviation and min–max values in parentheses. The calculation of prevalence values (%) follows the definition by Bush *et al*. (1997), and the density values were semiquantitatively evaluated by applying a scale from ‘1 + ’ representing the lowest 1 + and ‘ + + + + + +’ representing the highest 6 + density, a methodology modified from 200 ×  magnification by Gürkanlı *et al*. (2018). The density of infection categorized according to the mean and range of myxosporean parasites in parentheses were determined as 1 + (1–9), 2 + (10–19), 3 + (20–29), 4 + (30–39), 5 + (40–49) and 6 + (>50).

### Molecular analyses

To extract total genomic DNA from *Ortholinea*-infected host tissues of *Gobius niger*, *Symphodus cinereus* and *E. engrasicolus* of Okkay and Özer (2020) and *Neogobius melanostomus* of the present study, an Invitrogen PureLink^®^ Genomic DNA Mini Kit (USA) was employed. Extractions were performed according to the manufacturer's instructions and the DNA was hidden at −20 °C before use. To construct phylogenies, SSU rDNA was used as molecular marker. Amplification of the gene was carried out using primers of both MyxospecF (Fiala, 2006) and 18r (Whipps *et al*., 2003). PCR amplifications were made using a Techne (TC-Plus) thermal cycler with the following procedure; 3 min of initial denaturation at 95 °C, followed by 40 cycles of denaturation at 94 °C for 1 min, annealing at 51 °C (−0.1 °C per cycle) for 1 min, and extension for 1.5 min at 72 °C. The final extension was facilitated at 72 °C for 10 min. For all PCR amplifications, a 50 *μ*l reaction mixture was prepared with GoTaq^®^ Colorless Master Mix 2× (Promega, Madison, U.S.A.), 0.5 pmol (final concentration) of each primer (Oligomer), genomic DNA<1 *μ*g and sterile ddH_2_O (up to 50 *μ*l). For electrophoresis (to check both genomic DNA and PCR products), 1% agarose gel prepared in 1× TBE buffer was used and visualizations of the gels were performed with the photo print imaging system (Vilber Lourmat, France). Nucleotide sequencings were performed commercially by Macrogen-Europe from both strands with the same primers used for PCR amplifications. Verification and assemblage of nucleotide sequencings were made with Software BioEdit (Hall, 1999). For phylogenetic constructions, a data set was prepared in the light of available literature and also according to the results of BLAST (Basic Local Alignment Search Tool, https://blast.ncbi.nlm.nih.gov/Blast.cgi) search. Multiple nucleotide sequence alignment of the data set was performed with ClustalX (Thompson *et al*., 1997). Phylogenetic constructions were made using GTR + I + G (I: 0.312; G: 0.581) and TPM2 + I + G (I: 0.285; G: 0.533) evolutionary models that have been suggested by Akaike information criterion (Akaike, 1974) and Bayesian information criterion tests, respectively. These tests were performed using jModelTest v. 0.1 package program (Guindon and Gascuel, 2003; Posada, 2008). To construct phylogenies, maximum-likelihood (ML), neighbour-joining (NJ) (Saitou and Nei, 1987) and maximum-parsimony (MP) (Eck and Dayhoff, 1966; Fitch, 1977) methods were applied. Software program PAUP* v. 4.0b10 was implemented using both NJ and MP analyses (Swofford, 1998). A heuristic search approach with a TBR swapping algorithm (10 incidental repetitions) was applied for MP analysis. The software program PhyML 3.0 (Guindon and Gascuel, 2003) was employed for ML analysis. Bootstrap tests were performed with 10 000 replicates for NJ and 1000 replicates for MP and ML analyses (Efron, 1982; Felsenstein, 1985). BioEdit was used to resolute binary nucleotide sequence similarities. Genetic distances among genotypes, corrected in accordance with the previously mentioned evolutionary models, were computed using PAUP.

Our new 18S rDNA genotypes have been deposited in GenBank under accession numbers OR884251-OR884254 (Table 1).

**Table 1.** Source information of *Ortholinea* isolates obtained in this study and Myxozoan species obtained from NCBI (given with references) for phylogenetic analyses

Species	Host	Tissue Origin	Country	GenBank Acc. No.	Source
**AO-32** (*Ortholinea hamsiensis* n. sp.)	*Engraulis engrasicolus*	Urinary bladder	Türkiye	OR884251	Present study
**AO-81** (*Ortholinea gobiusi*)	*Neogobius melanostomus*	Kidney	Türkiye	OR884254	Present study
**AO-35** (*Ortholinea gobiusi*)	*Gobius niger*	Kidney	Türkiye	OR884252	Present study
**AO-54** (*Ortholinea divergens*)	*Symphotus cinereus*	Kidney	Türkiye	OR884253	Present study
*Ortholinea auratae*	*Sparus aurata*	Urinary bladder	Portugal	KR025868	Rangel *et al*. (2015)
*Ortholinea argusi*	*Scatophagus argus*	Urinary bladder	Malaysia	MH197371	Samshuri *et al*. (unpublished)
*Ortholinea concentraca*	*Acanthistius patachonicus*	Urinary bladder	Argentina	MH793352	Alama-Bermejo *and Hernandez-Orts* (2018)
*Ortholinea labracis*	*Dicentrarchus labrax*	Urinary bladder	Portugal	KU363830	Rangel *et al*. (2017)
*Ortholinea lauquen*	*Galaxias maculatus*	Kidney	Argentina	MN128729	Alama-Bermejo *et al*. (2019)
*Ortholinea mullusi*	*Mullus barbatus*	Urinary bladder and kidney tubules.	Türkiye	MF539825	Gürkanlı *et al*. (2018)
*Ortholinea nupchi*	*Paralichthys olivaceus*	Urinary bladder	South Korea	MW540886	Shin *et al*. (2023)
*Ortholinea orientalis*	*Sprattus sprattus*	Ureters	Denmark	HM770872	Karlsbakk and Køie (2011).
*Ortholinea scatophagi*	*Scatophagus argus*	Urinary bladder	India	MN310514	Chandran *et al*. (2020)
*Ortholinea* sp. RT_1	*Rhizoprionodon terraenovae*	Kidney	USA, Ca	MK937851	Lisnerová *et al*. (2020)
*Ortholinea* sp. JL-2021	*Alosa pseudoharengus*	Kidney	USA, NJ	MZ474836	Friend *et al*. (2021)
*Hoferellus alosae*	*Alosa alosa*	Kidney	France	KU301052	Wuennemann *et al*. (2016)
*Hoferellus carassii*	*Carassius gibelio*	Urinary bladder	Czech Rep.	KU141400	Alama-Bermejo *et al*. (2016)
*Hoferellus cyprini*	*Cyprinus carpio*	Urinary bladder	Czech Rep.	KU141402	Alama-Bermejo *et al*. (2016)
*Hoferellus gilsoni*	*Anguilla anguilla*	-	N. Scotland	AJ582062	Holzer *et al*. (2004)
*Hoferellus gnathonemi*	*Gnathonemus petersii*	Kidney	Nigeria	KU141398	Alama-Bermejo *et al*. (2016)
*Hoferellus jutubensis*	*Ageneiosus inermis*	-	Brazil	MW540793	Pereira *et al*. (2022)
*Hoferellus* sp. K41	*Cyprinus carpio*	Kidney	Czech Rep.	KU141401	Alama-Bermejo *et al*. (2016)
*Myxidium streisingeri*	*Danio rerio*	-	USA	KM001688	Whipps *et al*. (2015)
*Myxobilatus gasterostei*	*Nais communis*	-	USA	EU861209	Atkinson and Bartholomew (2009)
*Zschokkella* sp.	*Anguilla anguilla*	-	N. Scotland	AJ581918	Holzer *et al*. (2004)

## Results

In the present study, only the kidney of the round goby, *N. melanostomus* was found to be infected by a species of the genus *Ortholinea* Shulman, 1962 (Myxozoa: Ortholineidae) based on the following distinguishing characteristics of the genus: (1) myxospore morphology, (2) dimensions of myxospore length and width, (3) morphology of polar capsules and dimensions of their length and width, (4) molecular level peculiarities. Myxospore morphology and morphometry of the examined parasites corresponded well with *O. gobiusi* that was presented in its previous reports from the same fish host. On the other hand, the infected tissue samples previously used by Okkay and Özer (2020) were revisited for the *Ortholinea* species reported from *E. engrasicolus,* and a more detailed investigation of 20 previously alcohol-preserved myxospores from infected urinary bladder together with molecular evaluation revealed a new *Ortholinea* species namely *O. hamsiensis* n. sp. The details of taxonomic summary, morphology and infection indices of both species are provided below;

### *Taxonomic summary of* Ortholinea gobiusi *Naidenova, 1968*

#### Phylum: Cnidaria Hatschek, 1888 Subphylum: Endocnidozoa Schuchert, 1996 Class: Myxozoa Grasse, 1970 Subclass: Myxosporea Bütschli, 1881 Order: Bivalvulidae Shulman, 1959 Suborder: Variisporina Lom and Noble, 1984 Family: Ortholineidae Lom & Noble, 1984 Genus: Ortholinea Shulman, 1962 **Name:** *Ortholinea gobiusi* Naidenova, 1968 (Fig. 1A,B) **Host:** *Neogobius melanostomus* (Pallas, 1814) round goby **Locality:** Sinop coasts of the Black Sea, Türkiye (42° 05′ 68″ N, 35° 10′ 55″ E) **Prevalence of infection:** 4.8% (6 females out of 103) **Density of infection:** 1–5 individuals in the field of view (1 + ) (200 ×  magnification) **Description of myxospores:** The characteristic feature is a round or mostly ovoid myxospores, and the myxospore surfaces have external striations (Fig. 1C). Two polar capsules of the parasite are rounded and positioned almost in opposite directions. Parasite individuals were detected in the presporogonic and sporogonic stages (Fig. 1D). All morphometric data of fresh myxospores are provided in Table 2.

### *Taxonomic summary of* Ortholinea hamsiensis *n. sp. (Fig. 1E–H)*

#### Phylum: Cnidaria Hatschek, 1888 Subphylum: Endocnidozoa Schuchert, 1996 Class: Myxozoa Grasse, 1970 Subclass: Myxosporea Bütschli, 1881 Order: Bivalvulidae Shulman, 1959 Suborder: Variisporina Lom and Noble, 1984 Family: Ortholineidae Lom & Noble, 1984 Genus: Ortholinea Shulman, 1962 **Type host:** *Engraulis encrasicolus* (Linnaeus, 1758) European anchovy **Type locality:** Sinop coasts of the Black Sea, Türkiye (42° 02’ 68” N, 35° 10’ 55”E) **Prevalence of infection:** 1.4% (2 females out of 72) **Density of infection:** 1–5 individuals in the field of view (1 + ) (200 ×  magnification) **Type material:** One holotype (MyxoOH 2023.1) and 1 paratype (MyxoOH 2023.2) were hidden at the Faculty of Fisheries and Aquatic Sciences Parasitological Collection of the Sinop University, Sinop, Türkiye **Etymology:** Parasite species is derived from the local fishery name in Türkiye ‘hamsi’ of the host, *E. engrasicolus*

### Description

#### Myxospores of Ortholinea hamsiensis n. sp.

**Figure 1.**
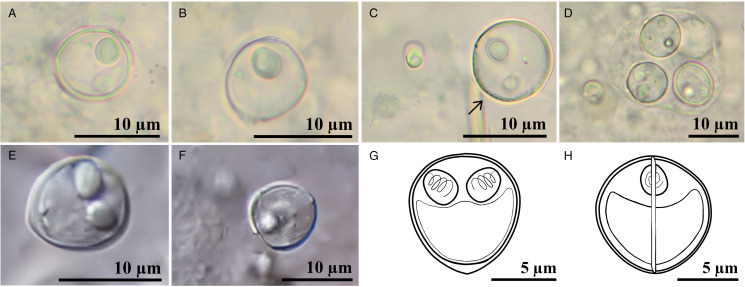
A fresh spore of *Ortholinea gobiusi*, (A) frontal view, (B) sutural view, (C) surface ridges indicating the presence of striations, (D) developmental sporogonic stage with developing myxospores; A myxospore of *O. hamsiensis* n. sp. observed by DIC objective, E. frontal view, F. sutural view; hand drawing of *O. hamsiensis* n. sp. G. frontal view, H. sutural view.

Immature and developing myxospores are oviform and slightly tapering down to the tip in the frontal view. Mature myxospores are subspherical with slight tapering down to the less pronounced tip in the frontal view and subspherical in the sutural view (Fig. 1E,F,G,H) with measurements of 9.1 ± 0.25 (8.8–9.9) *μ*m in length, 9.2 ± 0.11 (8.9–9.4) *μ*m in thickness and 8.4 ± 0.33 (8.2–9.1) *μ*m in width. Two polar capsules equal in size, located nearly at the higher 1/3 level of the myxospores, measuring 3.1 ± 0.11 (3.0–3.3) *μ*m in length and 2.7 ± 0.11 (2.6–2.9) *μ*m in width. The polar tubule had 3–4 coils.

**Table 2.** Site of infection, hosts, geographical localities and dimensions (*μ*m, ± SD) of species of the genus *Ortholinea* found in marine fish

Species	Spore body			Polar capsule			PTC	Site of infection	Prevalence (%)	Host species	Locality	Reference
	Length	Width	Thickness	Length	Width	Diameter						
*Ortholinea hamsiensis* n. sp.	9.1 ± 0.25 (8.8–9.9)	8.4 ± 0.33 (8.2–9.1)	9.2 ± 0.11 (8.9–9.4)	3.1 ± 0.11 (3.0–3.3)	2.7 ± 0.11 (2.6–2.9)	-	3–4	Urinary bladder	-	*Engraulis engrasicolus*	Black Sea coast Sinop, Türkiye	This study
*O. gobiusi*	8.6 ± 0.15 (8.3–8.8)	7.5 ± 0.18 (7.3–7.9)	6.7 ± 0.17 (6.5–7.0)	2.7 ± 0.16 (2.5–3.3)	2.1 ± 0.09 (2.0–2.3)	-	-	Kidney	4.8	*Neogobius melanostomus*	Black Sea coast Sinop, Türkiye	This study
*O. orientalis*	9.1 (9.0–9.4)	8.6 (8.2–8.9)	-	3.1 (3.0–3.3)	2.3 (2.2–2.4)	-	-	Urinary bladder	1.4	*Engraulis engrasicolus*	Black Sea coast Sinop, Türkiye	Okkay and Özer (2020)
*O. orientalis*	8.0 (7.7–8.3)	7.9 (7.7–8.2)	-	2.5 (2.3–2.7)	1.8 (1.7–2.0)	-	-	Urinary bladder	1.9	*Alosa immaculata*	Black Sea coast Sinop, Türkiye	Okkay and Özer (2020)
*O. divergens*	9.1 (8.2–9.6)	9.3 (8.5–9.8)	-	2.0 (1.8–2.2)	2.1 (1.8–2.3)	-	-	Kidney	33.3	*Symphodus cinereus*	Black Sea coast Sinop, Türkiye	Okkay and Özer (2020)
*Ortholinea* sp.	8.5 (8.3–9.3)	7.7 (7.4–8.9)	-	2.7 (2.0–2.9)	1.9 (1.9–2.2)	-	-	Kidney	9.09	*Gobius niger*	Black Sea coast Sinop, Türkiye	Okkay and Özer (2020)
*O. orientalis*	7.5–8.5	7.5–7.6	5.0	2.2–3.0	2.2–3.0	-	-	-	-	*Clupea harengus*	White Sea	Shulman and Shulman-Albova (1953)
*O. orientalis*	8.5–11.5	6.8–9.8	6.5–8.0	3.0–4.2	3.0–4.2	-	-	Urinary system	-	*Eleginus navaga*	White Sea	Shulman and Shulman-Albova (1953)
*O. orientalis*	7.3–9.0	6.3–7.2	-	2.8–3.2	1.8–2.0			Urinary system		*Clupea pallasi*	Sea of Okhotsk	Aseeva (2000)
*O. orientalis*	7.6–8.3	6.6–8.0	-	3.0–3.7	3.0–3.7	-	-	-	-	*Thyeragna chalcogramma*	Bering Sea	Aseeva (2002)
*O. orientalis*	9.3–10.3	8.6–9.3	-	3.5–4.0	2.7–3.5					*Eleginus gracilis*	Sea of Japan	Aseeva (2002)
*O. orientalis*	9.0 (8.5–9.2)	7.9 (7.7–8.0)	5.6 (4.9–5.8)	2.7 (2.3–2.9)	2.7 (2.3–2.9)	-	-	Ureters, renal tubules	20	*Clupea harengus*	The northern Öresund, Denmark	Karlsbakk and Køie (2011)
*O. orientalis*	9.0 (8.5–9.2)	7.9 (7.7–8.0)	5.6 (4.9–5.8)	2.7 (2.3–2.9)	2.7 (2.3–2.9)	-	-	Ureters, renal tubules	11	*Sprattus sprattus*	The northern Öresund, Denmark	Karlsbakk and Køie (2011)
*O. orientalis*	7.3 (7.1–7.5)	7.0 (6.9–7.2)	6.2 (6.0–6.4)	2.7 (2.6–2.9)	2.2 (2.1–2.3)	-	-	Urinary bladder	33.3	*Mullus barbatus ponticus*	Black Sea coast Sinop, Türkiye	Özer *et al*. (2015*a*)
*O. orientalis*	7.4 (7.2–7.5)	7.2 (7.0–7.4)	6.2 (6.1–6.4)	2.8 (2.7–3.0)	1.9 (1.8–2.0)	-	-	Urinary bladder	2.5	*Alosa tanaica*	Black Sea coast Sinop, Türkiye	Özer *et al*. (2015*a*)
*O. mullusi*	9.3 (9.0–9.7)	8.7 (8.2–9.3)	7.7 (7.5–7.9)	3.1 (3.0–3.2)	2.5 (2.4–2.6)	-	3-4	Urinary bladder, Kidney	24.5	*Mullus barbatus*	Black Sea coast Sinop, Türkiye	Gürkanlı *et al*. (2018)
*O. divergens*	10	-	8	-	-	4	20-25 *μ*	Urinary bladder	1/7, 1/23	*Blenius pholis, Crenilabrus melops)*	English Channel	Thélohan (1895)
*O. gobiusi*	8.3 (7.5–8.6)	7.2 (6.8–7.5)	-	4.9 (4.6–5.1)	2.0 (1.9–2.2)	-	-	Urinary bladder	4.1	*Neogobius melanostomus*	Black Sea coast Sinop, Türkiye	Özer *et al*. (2015*b*)
*O. gobiusi*	7.7–9.8	7.0–7.2	4.8–5.0	-	-	1.8–2.1	-	-	-	*Gobius ophiocephahis*	Black Sea, Sevastopol	Naidenova (1968)
*O. auratae*	9.0 (8.2–10.1)	8.3 (7.5–9.1)	7.2 (6.3–8.4)	3.2 (2.9–3.6)	2.7 (2.4–2.9)	-	-	-	-	*Sparus aurata*	the Atlantic coast, Portugal	Rangel *et al*. (2014)
*O. labracis*	7.6 (6.8–8.7)	7.2 (6.7–7.7)	6.5 (5.8–7.7)	3.0 (2.6–3.4)	2.4 (2.0–2.9)	-	4–5	Urinary bladder, Kidney	11.0	*Dicentrarchus labrax*	Alvor estuary, near the Atlantic coast, Portugal	Rangel *et al*. (2017)
*O. saudii*	10 (9–11)	12 (11–13)	-	-	-	4.5 (4.0–5.0)	-	Kidney	5.0	*Siganus rivulatus*	Red Sea coast, Jeddah, Saudi Arabia	Abdel-Baki *et al*. (2015)
*O. alata*	12.6	9.6	-	4.6	4.6	-	-	Kidney tubules	-	*Chaetodon rainfordi*	Australia	Kent and Moser (1990)
*O. striateculus*	10.1 (9.1–10.5)	10.0 (8.9–10.4)	-	3.5 (3.4–3.6)	2.9 (2.8–3.1)	-	5–7	Ureters	0.3	*Leptatherina presbyteroides*	Australia	Su and White (1994)
*O. scatophagi*	7.34 (6.22–8.71)	6.90 (5.90–8.21)	6.48 (6.11–6.88)	2.59 (1.66–3.23)	2.24 (1.27–2.98)	-	5	Urinary bladder, Ureter	70.14	*Scatophagus argus*	India	Chandran *et al*. (2020)

PTC, number of polar tubule coils. –: no data.

#### Differential diagnosis of Ortholinea hamsiensis n. sp.

A comparison of myxospore characteristics of presently reported new species with those of the original description of *O. gobiusi* from grass goby *Zosterisessor ophiocephalus* by Naidenova (1968) shows that myxospores of the new species in the present study are more subspherical and slightly tapering down to the less pronounced tip in frontal view, while myxospores of *O. gobiusi* are oviform and sharply tapering down to a pronounced tip with smaller myxospore dimensions. The same situation occurs when compared with *O. gobiusi* from the same fish host *N. melanostomus* inhabiting the same sampling locality (Özer *et al*., 2015*b*). Myxospores of *Ortholinea divergens* from *Parablennius sanguinolentus* (Özer *et al*., 2015*b*) are more rounded than those observed in the new species. The present species also differs from *O. divergens* in having smaller polar capsule dimensions. The shapes of the polar capsules of the new species and *O. mullusi* have different appearances, oval in the previous and pyriform in the latter species.

In the previous study by Okkay and Özer (2020), an *Ortholinea* species was found in the urinary bladder of *E. encrasicolus,* and based on the comparisons of myxospore morphology and morphometry with the previous wide range of host and geographical locality reports in the literature, they identified it as *O. orientalis*. However, a more detailed examination of these previously alcohol-preserved infected tissue myxospores of *Ortholinea* species, namely *O. hamsiensis* n. sp. in the present study, revealed that there were some differences when compared with the previous reports of *O. orientalis* from other host species inhabiting a wide range of geographical localities. The myxospores of the presently reported new species are subspherical with slight tapering down to the less pronounced tip in frontal view and *O. orientalis* has subspherical to triangular myxospores, with the broadest anterior and pointed posterior end together with a conspicuous triangular intercapsular process occurs at the anterior end of the myxospore (Karlsbakk and Køie, 2011). Myxospore dimensions of the new species are smaller than those of *O. orientalis* from navaga *Eleginus gracilis* (Tilesius, 1810) and *Eleginus nawaga* (Walbaum, 1792) but larger than those of *O. orientalis* from Atlantic herring *Clupea harengus* Linnaeus, 1758, Pacific herring, *Clupea pallasi* Valenciennes, 1847, Alaska Pollock, *Gadus chalcogrammus* Pallas, 1814, red mullet, *Mullus barbatus ponticus* Essipov, 1927, Black Sea shad, *Alosa tanaica* (Grimm, 1901) (Shulman and Shulman-Albova, 1953; Aseeva, 2000; Karlsbakk and Køie, 2011; Özer *et al*., 2015*a*). The polar capsules of the new species are larger than those of *O. orientalis* from *C. pallasii* and *E. nawaga*, *O. labracis* from the European seabass, *Dicentrarchus labrax* (Linnaeus, 1758), *O. scatophagi* from the spotted scat, *Scatophagus argus* (Linnaeus, 1766) but, smaller than those of *O. mullusi* from *M. barbatus ponticus, O. auratae* from the gilthead seabream, *Sparus aurata* (Linnaeus, 1758), *O. alata* from the northern butterflyfish, *Chaetodon rainfordi* McCulloch, 1923, and *O. striateculus* from silver fish, *Leptatherina presbyteroides* (Richardson, 1843) (Shulman and Shulman-Albova, 1953; Kent and Moser, 1990; Su and White, 1994; Özer *et al*., 2015*a*; Rangel *et al*., 2017; Gürkanlı *et al*., 2018; Chandran *et al*., 2020). *Ortholinea saudii* from marbled spinefoot *Siganus rivulatus* (Abdel-Baki *et al*., 2015) own too large polar capsules and myxospores compared to the new species in the present study.

#### Molecular analyses

**Figure 2.**
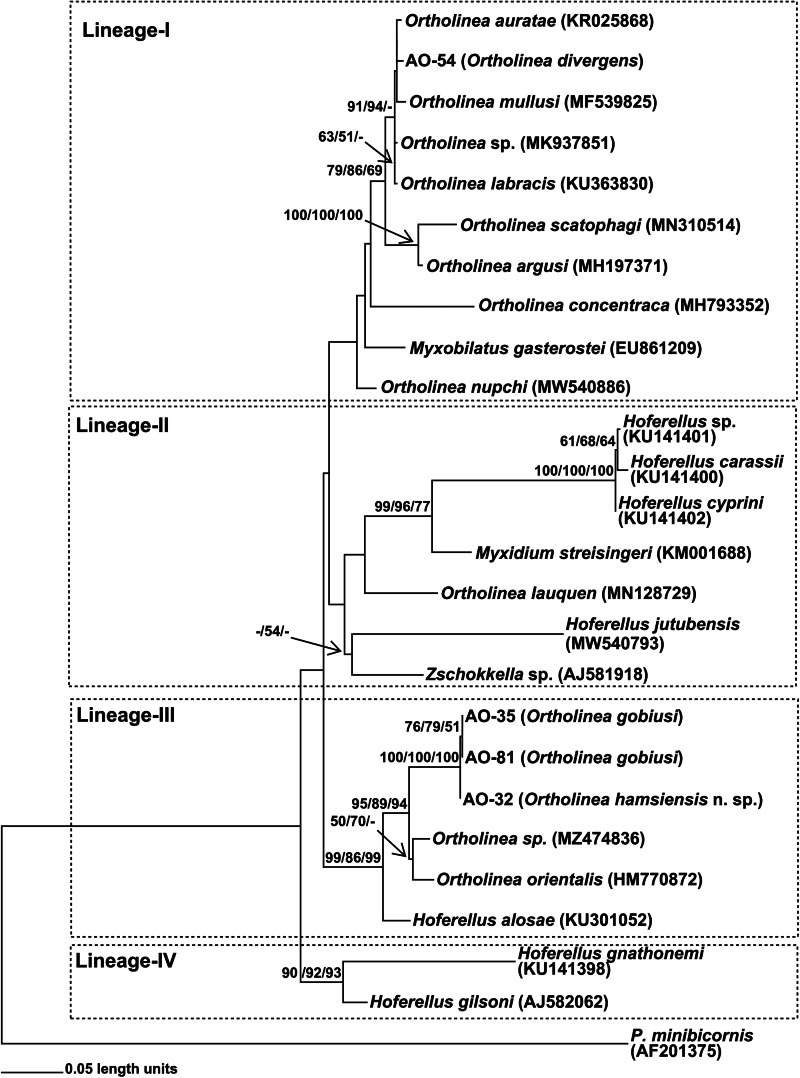
ML phylogram based on 18S rDNA nucleotide sequences of *Ortholinea* isolates obtained in this study (AO-32, AO-35, AO-54 and AO-81) and some closely related *Myxozoa* species downloaded from NCBI (given with GenBank accession numbers). On each related node bootstrap values (⩾50%) obtained from ML, NJ and MP analyses are stated with the given order. The tree is rooted with *Parvicapsula minibicornis* (Kent *et al*., 2000).

A total of 4 isolates of *Ortholinea* including AO-81 from *N. melanostomus*, AO-35 from *G. niger*, AO-54 from *S. cinereus* and AO-32 from *E. encrasicolus* were studied for molecular analysis. As a result of nucleotide sequencings, approximately 1700 bp of SSU rDNA were obtained from myxosporean specimens observed in infected host fish tissues. The codes for myxosporean genotypes obtained from different host fishes are given in Table 1. Concordant with the initial microscopic observations, BLAST searches associated all 4 genotypes obtained in this study with the genus *Ortholinea*, and thus, a data set was constituted with SSU rDNA sequences of available *Ortholinea* species together with some allied myxosporean species which are readily available in GenBank (Table 1). Of the 26 binomial species within the genus *Ortholinea*, only 11 of them had genetic records (SSU rDNA genotypes) in GenBank, thus, we were able to perform a genetic comparison with only this limited number of species. In addition, we also had to ignore 2 of the genetically available species, *O. saudii* and *O. amazonica*, due to their short SSU rDNA sequences in GenBank which caused a serious loss of genetic information in the aligned data set. As *Ortholinea* represents a paraphyletic lineage, we have also included several related species from the genera *Myxobilatus*, *Hoferellus* and *Myxidium*, in our data set. Phylogenetic analyses were performed over 981 (excluding gaps) aligned nucleotides with 392 segregated characters (509 substitution mutations). The ML trees created using GTR + I + G (I: 0.312; G: 0.581) and TPM2 + I + G (I: 0.285; G: 0.533) models were topologically similar, however, the ML tree with the initial model have suggested higher bootstrap values, thus considered in this study. The same situation was also observed in the NJ analysis. The Parsimony analysis that was conducted with 202 synapomorphic characters produced 48 single most parsimonious trees with 801 steps (CI: 0.635456; RI: 0.682609 and HI: 0.364544). In this study, the ML tree created using GTR + I + G model is given, additionally, bootstrap values obtained from NJ (with GTR + I + G model) and MP analyses have also been stated on each related node (Fig. 2).

On the phylogenetic trees 4 main lineages (-I, -II, -III and -IV) appeared (Fig. 2). However, the positioning of certain species, such as *Ortholinea nupchi*, *Myxobilatus gasterostei*, *Ortholinea concentraca*, *Hoferellus jutubensis* and *Zschokkella* sp. (AJ581918), displayed discrepancies among the trees generated using the ML, NJ and MP algorithms. Therefore, the placement of these species within a particular lineage lacks robust support, signified by the absence of bootstrap values in the phylogenetic trees. Conversely, the new genotypes examined in this study consistently demonstrated stable phylogenetic relationships within the respective lineages they were positioned.

Notably, the first 3 lineages proved to be paraphyletic, encompassing *Ortholinea* species along with those from other myxozoan genera such as *Myxobilatus* (Lineage-I), *Myxidium*, and *Zschokkella* (Lineage-II), as well as *Hoferellus* (Lineages-II and -III). On the other hand, Lineage-IV presented as monophyletic, comprising 2 *Hoferellus* species.

The genotype AO-54, originating from *S. cinereus* and initially identified as *Ortholinea divergens* based on morphological criteria in Okkay and Özer (2020), has placed within Lineage-I as sister to *Ortholinea auratae* (KR025868). The nucleotide sequence similarity and genetic distance between AO-54 and *O. auratae* were determined as 97.6% and 0.0337, respectively (see Supplementary Table). In all phylogenetic trees, *Ortholinea mullusi* consistently emerged as a sister to the lineage mentioned earlier, exhibiting 94.5% nucleotide sequence similarity and 0.05911 genetic distance with AO-54. On the other hand, bootstrap analyses unveiled a polytomy and suggested a single bootstrap value for the lineage encompassing AO-54, *O. auratae* and O. *mullusi*. For this very reason no bootstrap values were assigned to support the intralineage relationships within this group. Additionally, *O. labracis*, *O. scatophagi*, *O. argusi*, *O. concentraca*, *O. nupchi*, *Ortholinea* sp. (MK937851) and *Myxobilatus gasterostei* have also appeared within Lineage-I.

The other 3 novel genotypes obtained in this study, AO-32, AO-35 and AO-81, along with genotypes, *O. orientalis* (HM770872), *Ortholinea* sp. (MZ474836), and *Hoferellus alosae* (HM770872) collectively formed Lineage-III. The intraspecific relationships within this lineage appeared as consistent, as evidenced by significant bootstrap values observed at all nodes. Notably, genotypes AO-81 and AO-35, originating from *N. melanostomus* and *G. niger*, respectively, both belonging to the Gobiidae family, exhibited the highest nucleotide sequence similarity at 99.9%. Additionally, they displayed the lowest genetic distance, recorded at 0.00062, positioning them as closely related sisters. Genotype AO-32, which was previously designated as *O. orientalis* depending on morphological criteria (Okkay and Özer, 2020), appeared as closely related to the group mentioned earlier rather than *O. orientalis* genotype HM770872. In accordance with this, the nucleotide sequence similarity and genetic distance between AO-32 and AO-81 were 98.7% and 0.01120, additionally were 98.8% and 0.01057 between AO-32 and AO-35. However, contrasting figures emerged with AO-32 and the *O. orientalis* genotype HM770872 where these values were 90.2% and 0.09983 (Fig. 2, Supp. Table).

## Discussion

*Ortholinea* (Shulman, 1962), the target myxozoan genus in this study, is composed of coelozoic 26 binominal parasite species that infect mainly the urinary bladder of mostly marine and rarely freshwater fishes (Lom and Dyková, 2006; Shin *et al*., 2023). Despite its limited biological diversity when compared with some other myxosporean genera such as *Myxobolus*, *Myxidium,* etc., reported species from fishes of this genus reveal a worldwide distribution (Lom and Dyková, 1992; Rangel *et al*., 2014, 2015, 2017; Gürkanlı *et al*., 2018; Shin *et al*., 2023). Concordant with this data, 4 *Ortholinea* species (*O. divergens*, *O. gobiusi*, *O. orientalis, O. mullusi*) have been reported from the Black Sea coasts of Türkiye thus far (Özer *et al*., 2015*a*, 2015*b*; Gürkanlı *et al*., 2018).

Until the end of the 20th century, species identification processes within the genus *Ortholinea* have been solely based on morphological and morphometric features of myxospores just like in other myxosporean genera. However, only these morphological characters are limited in numbers and inadequate in variations, thus they are mostly insufficient to reveal the true genealogy of myxozoa (Fiala *et al*., 2015). Moreover, molecular phylogenetic studies depending on nucleotide sequences of SSU rDNA gene that were published in the last 2 decades clearly revealed the incongruences between molecular phylogeny and myxospore morphology-based classification systems in most myxosporean genera such as *Myxobolus, Henneguya, Sphaerospora*, *Myxidium*, *Zschokkaella* and *Chloromyxum*. All these genera appeared as polyphyletic or paraphyletic taxa in the phylogenetic trees (Kent *et al*., 2001; Fiala, 2006). Additionally, in a comprehensive study including the genera *Myxobolus, Kudoa, Henneguya, Chloromyxum, Sphaerospora, Sphaeromyxa* and *Myxidium*, it has been concluded that just restricted morphological characters are concordant with phylogeny obtained from SSU rDNA data because of the plasticity in myxospore morphology (Fiala and Bartošová, 2010). Likewise, the genus *Ortholinea* appeared as another paraphyletic myxosporean genus in phylogenetic studies since some species of *Acauda, Myxobilatus,* and *Hoferellus* genera appeared within the same lineage together with *Ortholinea* species (Rangel *et al*., 2014, 2017; Alama-Bermejo and Hernandez-Orts, 2018; Alama-Bermejo *et al*., 2019; Chandran *et al*., 2020). For this very reason, in today's systematic concept, molecular data are indispensable for the diagnosis of myxozoan specimens and the identification of new species. However, despite its necessity, the identification of the most valid *Ortholinea* species is still solely dependent on the morphological features and only 11 nominal species have molecular data (SSU rDNA nucleotide sequence) in GenBank. In this context, this study aims to obtain and phylogenetically analyse the SSU rDNA genotypes of some Black Sea-originated *Ortholinea* specimens reported in a previous study (Okkay and Özer, 2020) in addition to some original *Ortholinea* specimens obtained from *Neogobius melanostomus*.

*Ortholinea gobiusi* is one of the valid species that is lacking molecular data in the genus *Ortholinea*. This species was first identified by Naidenova (1968) from the urinary bladder of *Gobius ophiocephalus* in the northern Black Sea and for the next nearly 50 years no record was given for this species until 2015 when Özer *et al*., reported *O. gobiusi* from the urinary bladder of *Neogobius melanostomus* (Pallas, 1814) collected from the Sinop coast of Türkiye (southern Black Sea). According to the morphological features and morphometric data of myxospores, researchers identified and reported this species with 4.1% prevalence out of 76 fish samples (Özer *et al*., 2015*b*). Five years later, Okkay and Özer (2020) reported *Ortholinea* specimens similar to *O. gobiusi* from the kidney of another gobiid *Gobius niger*. However, they did not designate these specimens as *O. gobiusi* but named them as *Ortholinea* sp. particularly because of the differences in the polar capsule dimensions. As mentioned, none of these studies included molecular data. In this study, however, we identified some *Ortholinea* specimens from *N. melanostomus* using both morphological and molecular techniques (AO-81). Additionally, we also analysed *O. gobiusi* specimens (AO-35), previously reported as *Ortholinea* sp. from *G. niger* in Okkay and Özer (2020) from a molecular phylogenetic perspective. The morphological and morphometric data (Table 2) of the new *Ortholinea* specimens obtained from *N. melanostomus* were consistent with the *O. gobiusi* features reported by Özer *et al*. (2015*b*) and Naidenova (1968). Although the polar capsules of the *Ortholinea* specimens from *G. niger* were relatively smaller as mentioned earlier, other morphological and morphometric features were fitting well with *O. gobiusi* descriptive features (Naidenova, 1968; Okkay and Özer, 2020). As a result of molecular analyses, these 2 *Ortholinea* specimens showed SSU rDNA genotypes with 99.9% nucleotide sequence similarity and 0.00062 genetic distance. This much identity and low genetic distance between SSU rDNA genotypes of specimens, AO-35 and AO-81, clearly indicates that they belong to the same species (*O. gobiusi*). Morphological data of AO-35 (Okkay and Özer, 2020) and AO-81 (obtained in this study) also supported this inference (Table 2). As a result, depending on molecular and morphological data we designated AO-81 as *O. gobiusi*. In this study, we have provided the first molecular data, SSU rDNA sequences, of *O. gobiusi* (genotypes AO-81 and AO-35) and thus completing the deficiency in the description of this species.

In the present study, *O. gobiusi* was found in the kidney of *N. melanostomus* and this new information about its site of infection makes a new contribution to our current knowledge about its tissue selection that is being solely reported from the urinary bladder of its gobiid fish hosts. The infection prevalence in this study was determined as 4.8% and this value is very similar to that of its previous report 4.1% from the urinary bladder of the host fish from the same locality by Özer *et al*. (2015*b*).

Similar to *O. gobiusi*, another valid *Ortholinea* species that lacks molecular data is *O. divergens*. This species was initially identified and named as *Sphaerospora divergens* by Thélohan (1895) from the English Channel and subsequently transferred to the genus *Ortholinea* as the type species of the genus by Shulman (1962). As can be expected from a relatively old species, there is no type sample available for comparison. And over the years *O. divergens* reported from diverse geographical locations and hosts including; *Reinhardtius hippoglossoides* off the Labrador and Barents Sea, the North Atlantic Ocean, and the Bering Sea, the North Pacific Ocean (Wierzbicka, 1990*a*, 1990*b*, 1992), *Reinhardtius platessoides* and *Hippoglossoides platessides* in North Atlantic (Zubchenko, 1980, 1985), *Aidablennius sphynx*, *Diplodus annularis*, *Lipophrys pavo* (Syn. *Salaria pavo*), *Liza aurata* (Syn. *Chelon auratus*), *Parablennius sanguinolentus*, *P. tentacularis*, *Symphodus roissali*, *S. ocellatus*, *S. cinereus* and *Salaria pavo* in the northern Black Sea (Ukrainian coasts) (Yurakhno, 2009). This species has also been reported in the southern Black Sea (Turkish coasts) from *P. sanguinolentus* (Özer *et al*., 2015*b*) and *S. cinereus* (Okkay and Özer, 2020). In the present study, we phylogenetically analysed the nucleotide sequence of the SSU rDNA gene of *O. divergens* specimens (AO-54) from Okkay and Özer (2020). As a result of phylogenetic analyses, *O. divergens* turned out as a sister to *O. auratae* on 97.6% nucleotide sequence similarity and 0.0337 genetic distance. These 2 species also revealed significant morphological differences such as *O. divergens* possessing round or ovoid myxospores and pyriform polar capsules while *O. auratae* myxospores were ellipsoidal and spherical. Additionally, only 2–3 developing spores were observed in the plasmodium of *O. divergens*, whereas the size of the glycocalyx-like sheet-covered plasmodium was quite large inhabiting numerous developing myxospores of *O. auratae* in Rangel *et al*. (2014). The glycocalyx-like sheet covering the plasmodia is a rather evident characteristic differing from the other species. As a result, here in the present study, we have provided the first molecular data of *O. divergens* for international databases. These data are particularly important for future molecular-based systematic studies concerning the genetic boundaries, diversity and systematic conflicts of the genus *Ortholinea* since this species is the type species of the genus.

The most commonly reported member of the genus *Ortholinea* is *Ortholinea orientalis* which was initially identified by Shulman and Shulman-Albova (1953) from *Clupea harengus* and *Eleginus navaga* obtained from the White Sea. Subsequently, it has been reported from several fish species classified within the families Clupeidae, Gadidae, Alosidae and Mullidae which were collected from diverse localities including Denmark, Japan Sea, Fars East Sea and the Black Sea (Aseeva, 2000, 2002; Karlsbakk and Køie, 2011; Özer *et al*., 2015*a*). As can be expected from a myxozoan species reported from such different hosts and localities, morphometric data given in these studies were quite diverse and this situation makes species boundaries of *O. orientalis* quite wide and thus questionable. To test whether this prediction is valid, in the present study, we phylogenetically analysed *O. orientalis* specimens (AO-32) obtained from *E. encrasicolus*, a member of another family within Clupeiformes, collected previously off the Sinop coast of the Black Sea by Okkay and Özer (2020). Our results revealed that this prediction is valid, and AO-32 appeared as distantly related to *O. orientalis* genotype obtained from GenBank but instead was sister to *O. gobiusi* (Fig. 2). The nucleotide sequence similarity and genetic distance between AO-32 and *O. orientalis* specimen (HM770872) was only 90.2% and 0.09983 which were not enough to consider these specimens as a single species. Moreover, the nucleotide sequence similarities (98.7–98.8%) and genetic distances (0.01057–0.0112) between AO-32 and its sister species, *O. gobiusi* (AO-35 and AO-81), were also not sufficient to consider this specimen as *O. gobiusi*. The reason for this inference is; the intraspecific sequence similarities of valid *Ortholinea* species (*O. orientalis*: 99.6%; *O. labracis*: 100%; *O. auratae*: 99.8%; *O. concentrica*: 99.6%) are reported as higher than 99.5% (Gürkanlı *et al*., 2018). Additionally, the morphometric differences in the myxospore lengths of AO-32 and *O. gobiusi* specimens also supported this inference (Okkay and Özer, 2020; Table 2). As a result, depending on both morphological and molecular phylogenetic evidences provided above, we suggest AO-32 as a new species namely *Ortholinea hamsiensis* n. sp.

In conclusion, significant results obtained in this study can be summarized as follows; (i) a novel myxosporean species, namely *Ortholinea hamsiensis* n. sp. have been identified from the urinary bladder of *Engraulis engrasicolus,* (ii) the first molecular records for *Ortholinea divergens,* the type species of this genus, and (iii) the first molecular records for *O. gobiusi* have been provided. With this new data, the missing molecular parts of the descriptions of these 2 species have been completed and phylogenetic relationships of these species with other *Ortholinea* species have been revealed.

## Supporting information

Okkay et al. supplementary material Okkay et al. supplementary material

## Data Availability

Sequence data is available on the NCBI GenBank database. All other necessary data are included in the article and its supplementary materials.

## Supplementary material

The supplementary material for this article can be found at https://doi.org/10.1017/S0031182024000325.
