# Supplementary material for: New molecular evidence on the members of the genus Ortholinea (Cnidaria, Myxozoa) and the description of Ortholinea hamsiensis n. sp. infecting the urinary bladder of European anchovy Engraulis engrasicolus in the Black Sea
Source: Parasitology. 2024 Mar 6;151(5):485–94. doi: 10.1017/S0031182024000325 (PMC11106505; doi:10.1017/S0031182024000325)
Supplement: Okkay et al. supplementary material [file S0031182024000325sup001.docx]

**Supplementary Table.** A table presenting the pairwise nucleotide sequence similarities and genetic distances among the 18S rDNA genotypes employed for the phylogenetic analyses in this study.

|  |  | **1** | **2** | **3** | **4** | **5** | **6** | **7** | **8** | **9** | **10** | **11** | **12** | **13** |
| --- | --- | --- | --- | --- | --- | --- | --- | --- | --- | --- | --- | --- | --- | --- |
| **1** | ***O. argusi* (MH197371)** | - | 0.09396 | 0.10270 | 0.20610 | 0.20610 | 0.20611 | 0.08737 | 0.09325 | 0.12793 | 0.19727 | 0.07140 | 0.09437 | 0.19195 |
| **2** | ***O. auratae* (KR025868)** | 90.5 | - | 0.03370 | 0.18414 | 0.18414 | 0.18404 | 0.04612 | 0.04698 | 0.11527 | 0.19617 | 0.10231 | 0.04758 | 0.18127 |
| **3** | ***O. divergens***  **(AO-54)** | 89.7 | 97.6 | - | 0.19032 | 0.19032 | 0.19035 | 0.05629 | 0.05911 | 0.11981 | 0.19473 | 0.10828 | 0.05504 | 0.18564 |
| **4** | ***O. gobiusi***  **(AO-35)** | 79.9 | 81.4 | 81.0 | - | 0.00062 | 0.01057 | 0.18920 | 0.19315 | 0.18740 | 0.10202 | 0.20834 | 0.19692 | 0.08917 |
| **5** | ***O. gobiusi***  **(AO-81)** | 79.9 | 81.4 | 81.0 | 99.9 | - | 0.01120 | 0.19005 | 0.19230 | 0.18655 | 0.10129 | 0.20835 | 0.19606 | 0.08988 |
| **6** | ***O. hamsiensis* n. sp. (AO-32)** | 79.9 | 81.4 | 81.0 | 98.8 | 98.7 | - | 0.18996 | 0.19306 | 0.18826 | 0.09983 | 0.20832 | 0.19521 | 0.08561 |
| **7** | ***O. labracis* (KU363830)** | 91.2 | 95.4 | 94.4 | 81.1 | 81.0 | 81.0 | - | 0.04226 | 0.11506 | 0.19287 | 0.09292 | 0.04489 | 0.18322 |
| **8** | ***O. mullusi* (MF539825)** | 90.6 | 95.2 | 94.5 | 80.7 | 80.8 | 80.7 | 95.8 | - | 0.11212 | 0.19067 | 0.09720 | 0.04097 | 0.18527 |
| **9** | ***O. nupchi* (MW540886)** | 86.8 | 87.8 | 87.4 | 80.8 | 80.9 | 80.8 | 87.9 | 88.1 | - | 0.18825 | 0.12984 | 0.11585 | 0.18337 |
| **10** | ***O. orientalis* (HM770872)** | 80.7 | 80.7 | 80.9 | 90.0 | 90.0 | 90.2 | 81.0 | 81.0 | 80.9 | - | 0.20587 | 0.19262 | 0.06045 |
| **11** | ***O. scatophagi* (MN310514)** | 96.9 | 89.5 | 88.9 | 79.6 | 79.6 | 79.6 | 90.4 | 90.0 | 86.4 | 80.0 | - | 0.09765 | 0.19630 |
| **12** | ***Ortholinea* sp. (MK937851)** | 90.4 | 94.4 | 93.6 | 80.1 | 80.1 | 80.2 | 94.7 | 95.0 | 87.4 | 80.5 | 89.7 | - | 0.17845 |
| **13** | ***Ortholinea*. sp (MZ474836)** | 81.1 | 81.8 | 81.6 | 91.1 | 91.0 | 91.4 | 81.7 | 81.5 | 81.4 | 94.0 | 80.8 | 81.7 | - |
